# Production of brain-derived neurotrophic factor gates plasticity in developing visual cortex

**DOI:** 10.1073/pnas.2214833120

**Published:** 2023-01-12

**Authors:** Megumi Kaneko, Michael P. Stryker

**Affiliations:** ^a^Department of Physiology and Kavli Institute for Fundamental Neuroscience, University of California, San Francisco 94143

**Keywords:** brain-derived neurotrophic factor, monocular deprivation, amblyopia, recovery of function, TrkB receptor

## Abstract

We have previously shown that recovery of visual responses to a deprived eye during the critical period in mouse primary visual cortex requires phosphorylation of the TrkB receptor for BDNF [M. Kaneko, J. L. Hanover, P. M. England, M. P. Stryker, *Nat. Neurosci.* **11**, 497–504 (2008)]. We have now studied the temporal relationship between the production of mature BDNF and the recovery of visual responses under several different conditions. Visual cortical responses to an eye whose vision has been occluded for several days during the critical period and is then re-opened recover rapidly during binocular vision or much more slowly following reverse occlusion, when the previously intact fellow eye is occluded in a model of “patch therapy” for amblyopia. The time to recovery of visual responses differed by more than 18 h between these two procedures, but in each, the production of mature BDNF preceded the physiological recovery. These findings suggest that a spurt of BDNF production is permissive for the growth of connections serving the deprived eye to restore visual responses. Attenuation of recovery of deprived-eye responses by interference with TrkB receptor activation or reduction of BDNF production by interference with homeostatic synaptic scaling had effects consistent with this suggestion.

## Significance

We previously showed that recovery of responses in visual cortex to an eye deprived of vision during early life is prevented by blocking the TrkB receptor for brain-derived neurotrophic factor (BDNF), and BDNF production is known to be stimulated by neural activity. By demonstrating that BDNF production precedes rather than merely accompanies the increase in cortical responses that follows re-opening the deprived eye after monocular visual deprivation, we demonstrate here a causal role for BDNF production in gating the plasticity that underlies the recovery of responsiveness in the primary visual cortex.

Sensory experience strongly influences the maturation and refinement of neuronal connections in the mammalian cortex during postnatal development. In the visual system, depriving visual input by closing one eye (monocular deprivation: MD) for a few days during a critical period of heightened plasticity in early postnatal life leads to a pronounced decrease in the cortical representation of the deprived eye, which is observed both physiologically and anatomically (1, 2). A second manifestation of cortical plasticity is the recovery of cortical responsiveness to the closed eye when the vision in the eye is restored. Brain-derived neurotrophic factor (BDNF) has been proposed as a regulator of ocular dominance plasticity, as visual experience stimulates BDNF expression in the visual cortex (3, 4) and pharmacological interference of signaling mediated by TrkB, the receptor for BDNF, impairs the formation of ocular dominance columns (5, 6). Using a powerful chemical-genetic approach (7, 8), we previously demonstrated that TrkB signaling during the critical period is required for recovery, but not loss, of cortical responsiveness following MD in mice (9). In that study, we chose to examine recovery at 4 d after the deprived eyes were open, allowing the animals binocular vision (BV) after a similar duration of MD (9).

Here we sought to measure the relationship between the production of mature BDNF and the recovery of responses to the deprived contralateral eye in mouse primary visual cortex (V1). While recovery from the effects of MD requires function of TrkB receptor for BDNF, changes in BDNF production in V1 during the course of recovery have not been examined. As BDNF expression is stimulated by neural activity in the visual cortex (3, 4, 10–17) upregulation of BDNF is expected during recovery. It seemed therefore from the literature that BDNF production might follow and be a result of the increased cortical responses to the recovering eye’s pathways. What we found instead is that an increase in BDNF production precedes the increase in the efficacy of deprived-eye pathways by at least the shortest time interval that we measured, 6 h. BDNF production preceded the recovery of responses by a similar amount in two different circumstances—with rapid recovery during BV as well as with much slower recovery following reverse occlusion (RO). These findings are consistent with a causal role for BDNF in strengthening excitatory pathways in the cortex.

## Results

### Recovery of Deprivation-Induced Depression in Cortical Responses is Faster during BV than during RO.

**Fig. 1.**
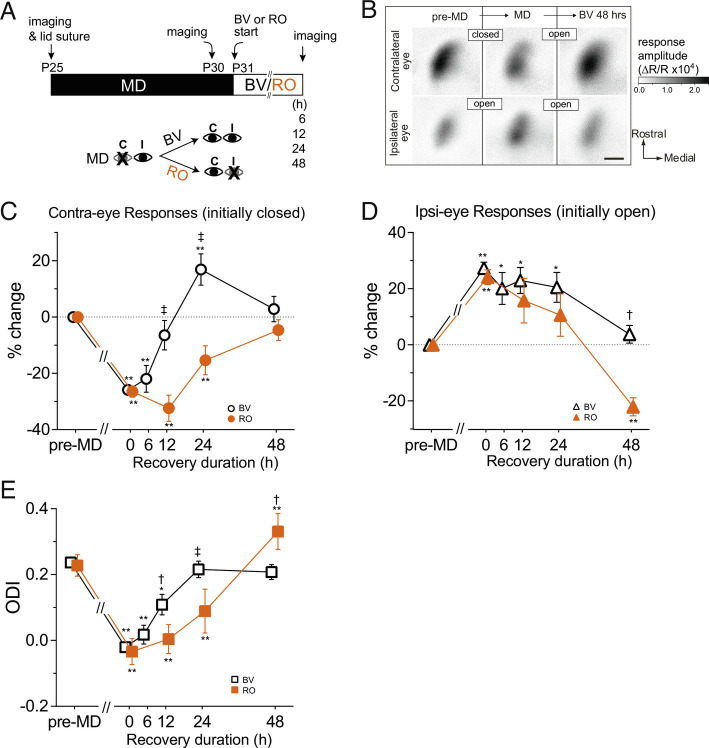
Faster recovery of deprived-eye responses by binocular vision than by reverse occlusion. (*A*) Experimental schedule for repeated optical imaging of intrinsic signals. MD: monocular deprivation of contralateral eye. BV: binocular vision, open symbols in *C*–*E*. RO: reverse occlusion, filled symbols in *C*–*E*. (*B*) Example of changes in response magnitude in an animal undergoing MD followed by recovery by BV. The gray scale represents the response magnitude as a fractional change in reflectance. Scale bar, 0.5 mm for all panels. (*C*) Changes in the response magnitude to the contralateral deprived eye (closed → open) during recovery. (*D*) Changes in the response magnitude to the ipsilateral eye (open → open for BV, open → closed for RO) during recovery. (*E*) Shift in ocular dominance during recovery calculated from data in *C* and *D*. Data in *C* and *D* are presented as % change from pre-MD baseline. Dotted lines in *C* and *D* represent the baseline level. All graphs show mean ± SEM. Sample size: BV-6 h (4), BV-12 h (5), BV-24 h (5), BV-48 h (5), RO-12 h (5), RO-24 h (5), RO-48 h (6). *P < 0.05, **P < 0.01 vs. pre-MD baseline, repeated measure ANOVA followed by multiple comparisons with Bonferroni’s correction. †*P *0.05, ‡*P *< 0.01 between BV and RO, one-way ANOVA followed by multiple comparisons with Bonferroni’s correction.

We examined recovery using chronic optical imaging of intrinsic signals in V1 during the developmental critical period to measure the magnitude of cortical responses to visual stimuli repeatedly in the same animals before eyelid suture, after 5 d of MD, and after various durations of either BV or RO (Fig. 1*A*). Previous studies showed recovery of ocular dominance by 4 d of BV (9) or 4 d of RO (18) following 4 to 6 d of MD during the critical period. Therefore, we examined the effects of BV or RO for a duration of 2 d or shorter (Fig. 1*A*). Note that different groups of mice were used for each time point because the necessity for short durations of recovery to compare RO and BV precluded repeated exposure of animals to anesthetics. Fig. 1*B* shows examples of intrinsic signal images after MD followed by 2 d of BV.

Recovery of closed-eye responses occurred faster with BV than with RO (Fig. 1*C*). Comparing responses to baseline levels measured before MD, closed-eye responses were first restored significantly after 12 h of BV (BV-12 h) nearly to baseline (−6.5 ± 10.4 % of baseline, *P* = 0.019 vs. MD, Fig. 1*C*) from their level immediately at the end of MD (−25.9 ± 5.9 (mean ± SD)% of baseline); whereas closed-eye responses during RO showed no significant recovery by 12 h (post-MD: −26.4 ± 7.5% of baseline; 12 h: −32.3 ± 9.3% of baseline). At BV-24 h, closed-eye responses were mildly but significantly increased beyond the baseline (16.9 ± 11.1% of baseline, *P* = 0.017 vs. baseline, Fig. 1*C*), returning to the baseline level at BV-48 h (2.9 ± 8.9% of baseline, *P* > 0.9, Fig. 1*C*). During RO, full restoration of previously closed eye responses was first seen at 48 h (RO-24 h: −15.4 ± 10.3% of baseline, *P* = 0.03; RO-48 h: −4.7 ± 7.4% of baseline, *P* = 0.07, *P* values are vs. baseline, Fig. 1*C*).

Changes in the magnitude of open-eye responses were different from and not strictly complementary to those of the closed eye. At BV-12 h and BV-24 h, open-eye responses were still at an elevated level similar to those observed immediately after MD (MD: 27.3 ± 8.6%; BV-12 h: 22.9 ± 9.3%, BV-24 h: 20.5 ± 10.6% of baseline, Fig. 1*D*).

These changes in response magnitudes to the closed and open eyes resulted in fast recovery of the ocular dominance index (ODI) during BV, which was already evident at BV-12 h (MD: −0.020 ± 0.036, BV-12 h: 0.109 ± 0.062), was complete by 24 h (0.216 ± 0.051), and did not further change at 48 h (0.208 ± 0.045, Fig. 1*E*).

Delayed recovery of ODI with RO (Fig. 1*E*) was a result of a slower increase in previously closed-eye responses, which seemed to take 48 h for full recovery to the baseline (Fig. 1*C*). At this time point, responses to the previously open, newly closed, eye were depressed (−22.1 ± 7.2% of baseline, Fig. 1*D*), resulting in significant ocular dominance shift toward the previously closed eye (RO-48 h: 0.331 ± 0.055, baseline: 0.218 ± 0.032, *P* < 0.05, Fig. 1*E*).

These results show that responses to the previously closed eye recovered faster when both eyes were open than when the occlusion was reversed following MD. Additional statistical comparisons are: MD vs. BV-6 h: n.s., *P* > 0.99, 95%CI: −15.3 to 6.6; MD vs. BV-12 h: *P* = 0.015; MD vs. BV-24 h: *P* = 0.0063; MD vs. RO-12 h: n.s., *P* >0.99, 95%CI: −2.4 to 14.6; MD vs. RO-24 h: n.s., *P *= 0.19.

### Requirement of TrkB Kinase Function for Rapid Recovery.

Transgenic mice (TrkB^F616A^) in which a phenylalanine-to-alanine substitution within the ATP binding pocket of the kinase renders the receptor sensitive to specific inhibition by small molecule 1NMPP1, a derivative of the general kinase inhibitor PP1, allow for specific and temporally controlled inactivation of TrkB (8). Using these mice, we have shown that tyrosine kinase activity of the TrkB neurotrophin receptor is required for full recovery from the effects of MD during 4 d of BV (9). Because we found that recovery occurred much faster as described above, we examined the effects of TrkB inactivation during 2 d of BV or RO.

**Fig. 2.**
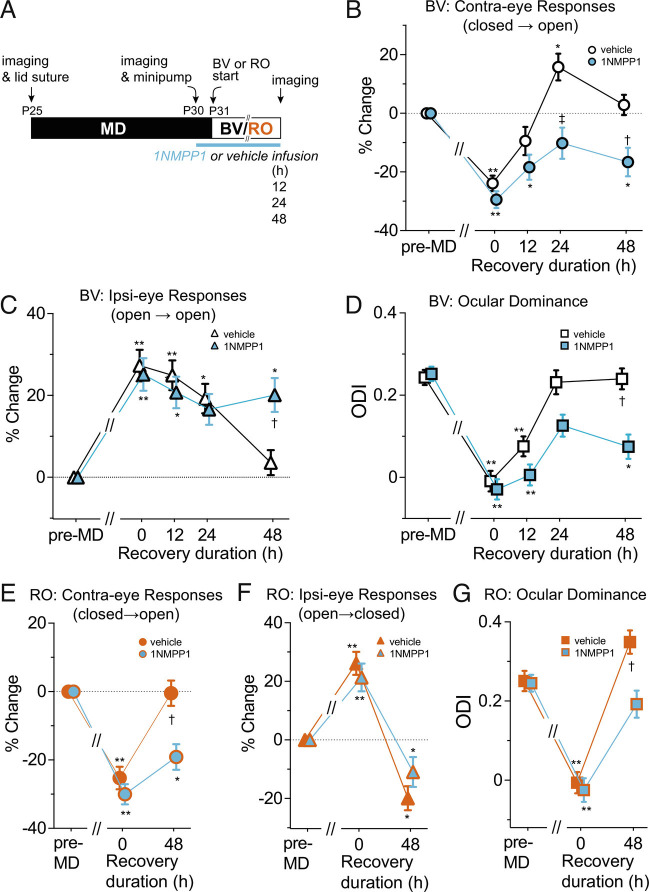
Effects of blocking TrkB receptor function on the time course of recovery. (*A*) Experimental schedule. (*B*–*D*) Recovery by BV in TrkB^F616A^ mice treated with vehicle or 1NMPP1 infusion. %Change in response magnitude to contralateral deprived-eye (closed → open) (*B*) and to ipsilateral eye (open → open) (*C*), and shift in ocular dominance index (*D*). Sample size for vehicle-treated animals: 12 h (4), 24 h (5), 48 h (5); for 1NMPP1-treated animals: 12 h (4), 24 h (5), 48 h (5). (*E*–*G*) Recovery by reverse occlusion in TrkB^F616A^ mice treated with vehicle or 1NMPP1 infusion, presented as %change in the response magnitude to the contralateral deprived eye (closed → open) (*E*) and to the ipsilateral eye (open → closed) (*F*); and shift in ocular dominance index (*G*). *P < 0.05, **P < 0.01 vs. pre-MD baseline, repeated-measure ANOVA followed by multiple comparisons with Bonferroni’s correction. †*P *< 0.05, ‡*P* < 0.01 between vehicle- and 1NMPP1-treated groups, one-way ANOVA followed by multiple comparisons with Bonferroni’s correction.

TrkB^F616A^ homozygous mice were given 1NMPP1 or vehicle solution via osmotic minipump throughout the duration of BV (12, 24, or 48 h) or RO (48 h) (Fig. 2*A*), to induce at least partial inactivation of TrkB receptors. Consistent with our previous result (9) for 4d-BV, administration of 1NMPP1 greatly reduced the restoration of cortical responses to the previously closed eye during 48 h of BV (vehicle: 2.9 ± 5.9% of pre-deprivation baseline, 1NMPP1: −16.7 ± 8.4% of baseline, *P* < 0.05) (Fig. 2*B*). However, closed-eye responses did increase modestly between 0 h and 24 h (0 h: −29.4 ± 5.8% of baseline, 24 h: −10.2 ± 9.3% of baseline, *P* < 0.05). Responses to the previously open eye in 1NMPP1-treated mice did not change during the whole period of 48 h BV; that is, they stayed at the same elevated level of MD (Fig. 2*C*). As a result, the ocular dominance was partially restored at 24 h (0 h: −0.03 ± 0.06, 24 h: 0.13 ± 0.05, *P* < 0.05) but fell back to a level similar to that of MD at 48 h (0.07 ± 0.05) (Fig. 2*D*).

Similar to its effect during BV, administration of 1NMPP1 also inhibited recovery of responses through the previously closed eye during RO (vehicle: −0.47 ± 7.4%, 1NMPP1: −19.0 ± 7.5%, *P* < 0.01) (Fig. 2*E*). In contrast, it did not inhibit the decrease in responses to the previously open, newly closed eye (vehicle: −19.9 ± 8.2% of baseline, 1NMPP1: −11.0 ± 10.2% of baseline, *P* > 0.05) (Fig. 2*F*). Consequently, ocular dominance in TrkB inactivation group was shifted toward the previously closed eye during RO approximately to the baseline pre-deprivation level (1NMPP1: baseline: 0.25 ± 0.04, RO: 0.19 ± 0.07, *P* > 0.05), but was significantly less shifted compared to the control group (vehicle: baseline 0.25 ± 0.05, RO: 0.35 ± 0.06) (Fig. 2*G*). This lack of effect of TrkB inactivation on depression of responses to the newly closed eye is consistent with our previous observation that it was not required for decrease in closed-eye responses during MD (9).

### Upregulation of BDNF Protein during Physiological Recovery.

**Fig. 3.**
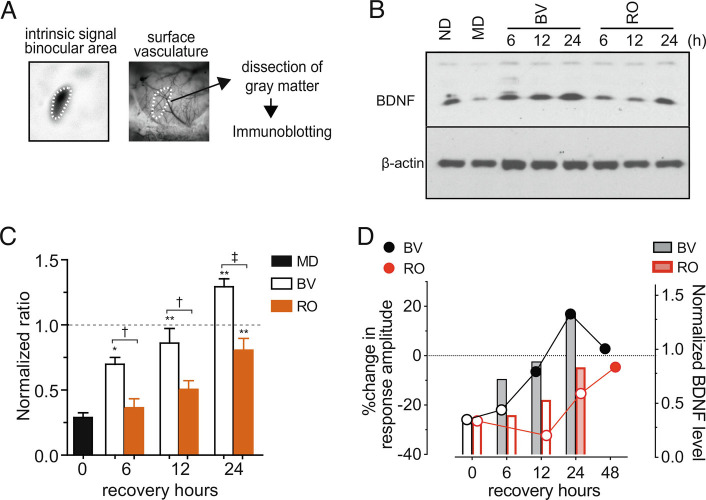
Rapid upregulation of BDNF in the primary visual cortex during recovery by binocular vision. (*A*) Dissection of the binocular visual cortex that has been localized using the surface vascular pattern and the intrinsic signal image. (*B*) An example of the immunoblot for mBDNF. Beta-actin was used as a loading control. (*C*) Quantification of density of the immuno-blot. The magnitude of the BDNF signal was first normalized with the corresponding beta-actin signal and then normalized with the magnitude of no deprivation (ND) control in the same experiment. Data are presented as mean ± SEM. Sample size is five pairs for each condition. MD: monocular deprivation, BV: binocular vision following 5 d of MD, RO: reverse occlusion following 5 d of MD. *P < 0.05 and **P < 0.01 vs. MD; †*P *0.05 and ‡*P *< 0.01 between BV and RO; all statistical analyses were done by one-way ANOVA followed by multiple comparisons with Bonferroni’s correction. (*D*) Comparison between physiological recovery of deprived-eye responses (on the left ordinate, from Fig. 1*C*) and mBDNF upregulation (on the right ordinate, from Fig. 3*C*) during BV or RO following MD. Filled symbols and open symbols represent statistically significant and insignificant changes, respectively, from the post-MD, 0 h recovery point.

Neural activity-dependent expression of BDNF has been extensively documented (19–22). Its expression in the visual cortex is modulated by visual experiences and is down-regulated during MD (3, 4, 10, 12–14). We examined the changes in BDNF protein level within the binocular area of V1 during BV or RO (Fig. 3*A*). We used Western blot analysis (Fig. 3*B*) because it allows distinction between mature BDNF (mBDNF) and pro-BDNF; the former, a proteolytic product of the latter, is the high-affinity ligand for TrkB (23).

Consistent with previous studies, mature BDNF protein level was significantly downregulated in the binocular area immediately after 5 d of MD (29.0 ± 5.1% of ND control, *P* < 0.05 vs. ND). Upregulation of mBDNF during the recovery period occurred faster with BV than with RO (Fig. 3*C*). There was a partial but significant increase from MD level already at BV-6 h (69.9 ± 5.2% of ND control, *P* < 0.05 vs. MD) and mBDNF reached almost to the ND level at BV-12 h (86.1 ± 11.3%, *P* > 0.05 vs. ND). Thereafter, mBDNF increased slightly but significantly beyond the ND level at BV-24 h (129.3 ± 6.1%, *P* < 0.05 vs. ND). Under RO, the first significant increase in mBDNF was measured at 24 h, by which time it had reached nearly normal levels (80.1 ± 9.1% of ND control, *P* < 0.01 vs. MD); levels at 6 h and 12 h were little changed from their values immediately after MD (6 h: 36.4 ± 6.9%, 12 h: 50.6 ± 9.5%, both *P* > 0.05 vs. MD).

How do changes in the BDNF level relate to functional recovery? At BV-6 h, mBDNF had more than doubled from that after MD (Fig. 3*D*), whereas the deprived-eye responses had increased by less than 5% and were on average still at MD level (Fig. 3*D*). During RO, a significant increase in mBDNF first appeared at RO-24 h (Fig. 3*D*), but the responses of the initially deprived eye had not yet increased significantly (Fig. 3*D*). Thus, during both BV and RO, the increase in mature BDNF protein level preceded functional recovery of the responses to the previously closed eye.

### Homeostatic Synaptic Scaling during MD Contributes to Rapid Recovery by BV.

When overall cortical activity is reduced, the strength of excitatory synapses increases, a process referred to as homeostatic synaptic scaling. This process is mediated at least partly by signaling through tumor necrosis factor alpha (TNFα) (24, 25). During MD, the initial decrease in closed-eye responses lowers overall cortical activity, thereby engaging TNFα-dependent homeostatic scaling to increase responses to the open eye (24). Homeostatic scaling also works on closed-eye responses to increase them to a lesser degree. We tested whether such a homeostatic increase in responsiveness through TNFα signaling contributes to the faster recovery of the previously closed-eye responses and changes in ocular dominance.

**Fig. 4.**
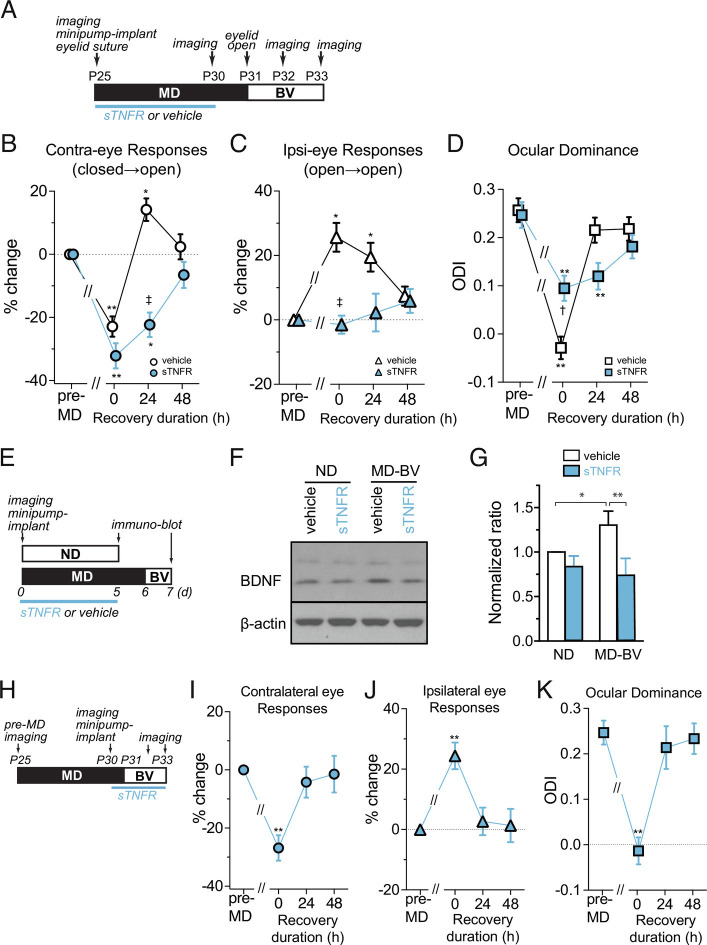
TNFα signaling during MD contributes to rapid functional recovery and BDNF upregulation. (*A*) Experimental schedule. (*B*–*D*) Changes in responses to the contralateral eye (closed → open, *B*); to the ipsilateral eye (open → open, *C*); and in ocular dominance (*D*) during recovery by binocular vision (BV) following 6 d of MD. Dotted lines in *B* and *C* represent baseline level. The response magnitude is shown as % change from pre-MD baseline. Data are presented as mean ± SEM. Sample size: vehicle (5, open symbols in black), soluble TNF receptor (sTNFR) (5, symbols filled with blue). *P < 0.05 and **P < 0.01 vs. pre-MD baseline, repeated-measure ANOVA followed by multiple comparisons with Bonferroni’s correction. †*P *0.05 and ‡*P* < 0.01 between vehicle and sTNFR groups, one-way ANOVA followed by multiple comparisons with Bonferroni’s correction. (*E*–*G*) Effects of sTNFR infusion on mBDNF level. (*E*) Experimental schedule. (*F*) An example of the mBDNF immunoblot. (*G*) Quantification of the mBDNF level. Signal strength for mBDNF was first normalized to the corresponding beta-actin signal and then normalized to that of no-deprivation vehicle control in each blot. *P < 0.05 and **P < 0.01, one-way ANOVA followed by multiple comparisons with Bonferroni’s correction. (*H*–*K*) Effects of sTNFR infusion during recovery by BV. sTNFR infusion was started immediately after post-MD imaging, followed 1 d later by the start of binocular vision (*H*). Changes in responses to the contralateral deprived eye (closed → open, *I*) and to the ipsilateral eye (open → open, *J*), and shift in ocular dominance (*K*). Data are shown as mean ± SEM. **P < 0.01 vs. pre-MD baseline, one-way ANOVA followed by multiple comparisons with Bonferroni’s correction.

Consistent with our previous study (24), partial blockade of TNFα signaling by cortical infusion of soluble TNF receptors (sTNFRs) during 5 d of MD (Fig. 4*A*) almost completely suppressed the normal increase in open-eye responses and caused a slightly larger decrease in closed-eye responses, resulting in a much smaller ODI shift compared to vehicle control (points at 0 h in Fig. 4 *B*–*D*). In these animals, 24 h of BV produced no significant change either in responses to the previously closed eye or in ocular dominance. Instead, it took 2 d for significant changes to occur (Fig. 4 *B*–*D*). This effect of blocking TNFα signaling was to reduce the strength of the synapses that would otherwise have increased in strength, as they did with vehicle infusion. The synapses left less powerful by the blockade of TNFα signaling would presumably produce less of the cortical activity that stimulates the secretion of mBDNF.

Indeed, the delayed recovery of function under TNFα blockade was accompanied by reduced mBDNF upregulation. In mice treated with sTNFR infusion, the levels of mBDNF after 24 h of BV were significantly lower than vehicle control animals and were similar to those in non-deprived mice infused with vehicle solution (BV-sTNFR:73 ± 19% of ND-veh). In contrast, in animals treated with vehicle solution, mBDNF levels after 24 h of BV were significantly increased to 130% of the levels in non-deprived vehicle control animals (Fig. 4 *F* and *G*), similar to the increased level we had measured in animals after 24 h of BV with no cortical infusion, as shown in Fig. 3.

A potential complication is that sTNFR might have not cleared completely when BV was started so that residual sTNFR might have affected recovery. However, this possibility is unlikely because infusion of sTNFR starting 1 d before and throughout the duration of 48 h of BV (Fig. 4*H*) had no detectable effect on recovery of responses, which was similar to that in control BV animals with no infusion (Fig. 4 *I*–*K*). Thus, an increase in synaptic strength during deprivation, mediated by TNFα signaling, is necessary for both the spurt of BDNF production and for the rapid recovery of cortical responses during the first 24 h of restored BV.

This finding provides further support for the coupling between the production of mBDNF and recovery of cortical responses to the deprived eye.

## Discussion

In nearly pure neuronal cultures studied in vitro, the formation of new synapses and the activity-dependent strengthening of existing ones requires signaling by BDNF on its principal receptor, TrkB (22, 26–32). In vivo, neural activity has long been known to stimulate the production of mature BDNF (3, 4, 10–17, 33). To determine whether the recovery of responses after the cessation of MD to the re-opened, formerly deprived, eye requires BDNF-TrkB signaling, and is therefore likely to rely on the strengthening of existing synaptic connections and the formation of new ones, we used a chemical-genetic approach (9). We studied recovery in mice in which TrkB receptors had been engineered to be susceptible to a small-molecule inhibitor (7, 8). Application of the inhibitor in these mice blocked the recovery of deprived-eye responses, as well as the apparently homeostatic reduction in the responses to the open eye. These findings provided strong evidence that BDNF secretion mediated an essential step in the recovery of deprived-eye responses.

To test this hypothesis further, here we examined the time course of the appearance of mature BDNF in relation to the recovery of deprived-eye responses after the re-opening of the deprived eye. If BDNF plays a causal role in recovery, then it should appear before responses recover when BDNF production and responses are examined with sufficient time resolution. Here we studied the recovery of deprived-eye responses under two different conditions: one in which the deprived eye was opened while the fellow eye remained open; and a second, referred to as reverse occlusion, in which the fellow eye was deprived of vision by lid suture at the time the initially deprived eye was opened. The former condition produces rapid recovery, in less than 24 h; recovery in the latter condition is much slower, presumably because neither eye drives the cortex well immediately after reverse suture. In both cases, the appearance of mature BDNF as measured with Western blots preceded recovery of deprived-eye responses by 6 h or more, consistent with a role in promoting the growth of synapses serving that eye’s pathways. In the case of the more rapid recovery, BDNF levels and responses actually overshot before returning to normal levels.

A limitation of the current findings is that they do not reveal which cells are making the BDNF whose production is increased by opening the deprived eye, and they also do not reveal on which cells the increased BDNF is acting directly, although (34) provides evidence that the excitatory cells of the cortex are one of the sources. The findings do not reveal whether BDNF acts on local Hebbian mechanisms of synaptic plasticity or more globally. Hence the conclusion to be drawn from the present findings is that BDNF production acts permissively to permit the plasticity responsible for recovery of closed-eye responses at sites and by mechanisms not specified. The conclusion is supported by the fact that TrkB receptor activation is necessary for recovery (9 and Fig. 2) and that BDNF production precedes recovery in several different conditions (Figs. 3 and 4).

### A Model of the Role of BDNF Signaling in MD and Recovery.

These and other findings support the following model of the events responsible for MD and recovery. Occluding the vision of the contralateral eye, which is the dominant eye in the mouse, dramatically reduces cortical activity and leads to the loss of synapses serving that eye (35). The reduction in cortical activity stimulates homeostatic synaptic scaling, which makes the remaining synapses stronger through a process mediated by TNFα signaling, leading to some recovery of cortical activity, a large increase in responses to the intact fellow eye, and a smaller increase in the responses to the deprived eye. When the deprived eye is re-opened, while its pathway’s synapses are stronger than they would otherwise be by virtue of synaptic scaling, they are also fewer than they once were. However, opening that eye, and leaving the fellow eye open as well, immediately increases cortical activity to a great extent, leading to a spurt of mature BDNF production. The BDNF acts on TrkB receptors to allow the strengthening of existing synapses serving the deprived eye and to promote the formation of new ones in the newly active pathways serving the formerly deprived eye.

In contrast, during RO when the fellow (ipsilateral) eye is closed at the time that the deprived (contralateral) eye is re-opened, cortical activity is not increased; at the end of 4 d of MD in the mouse, the two eyes drive the cortex more or less equally well, so that closing one when the other is opened does not change the total excitation of the cortex. In this case, the production of mature BDNF is not stimulated and remains at its constitutive level. This level of BDNF permits the strengthening and addition of synapses in the pathway serving the initially deprived (contralateral) eye to proceed only slowly, leading to a protracted recovery of its responses. Note that this process has positive feedback: as the deprived eye synapses strengthen, it activates the cortex more powerfully so that by 24 h of monocular recovery BDNF production reaches a level similar to that after 6 h of binocular recovery.

This model, which describes the results in Fig. 1, is supported by the results in Fig. 2, in which BDNF signaling is attenuated using the chemical-genetic inhibitor of its TrkB receptor. Recovery of deprived (contralateral) -eye responses during BV is dramatically slowed, and open (ipsilateral) -eye responses, which had increased during MD, remain elevated.

The rapid production of mature BDNF during BV, shown in Fig. 3, provides further support for this model of deprivation and recovery, as does its delayed appearance during RO.

The reduction of homeostatic synaptic scaling illustrated in Fig. 4 provides additional support for the model. Blocking synaptic scaling with sTNFR reduces both the increase in cortical activity triggered by BV immediately upon reopening the deprived eye and the consequent increase in the production of mature BDNF. The increase in responses to the deprived eye is delayed, presumably until constitutive BDNF production suffices for enough synapses serving the deprived eye to be strengthened or formed.

Of course, BDNF production is nowhere near the whole story. The role posited here for BDNF signaling does not specify which synapses will be strengthened. In this model, BDNF is thought merely to be permissive for the strengthening of synapses and the formation of new ones. The specification of which synapses are to be strengthened or added is presumably a result of normal Hebbian plasticity, with its conventional limits that keep it from running away completely.

The model as presented is abstract. The eyes do not project to the cortex, and within the cortex evidence indicates that plasticity is much more rapid in the upper layers than in the layers that receive the largest input from the lateral geniculate nucleus (36). So when we speak of strengthening the deprived eye pathways, those pathways span at least three synapses between the eyes and the site of the most rapid plasticity in the visual cortex. The abstract nature of this account is made further clear by the ongoing turnover of synapses in the primary visual cortex during the critical period, which encompasses the entire time course of the current study. The net effect of occluding the contralateral eye is a reduction in the number of synaptic boutons in layer 2/3 (35). Subsequent BV then restores the number of boutons to control levels.

With these limitations, however, the model of deprivation and recovery and the proposed role for BDNF signaling in that process has strong experimental support. The most important finding of this study is that the BDNF production precedes the increase in the efficacy of deprived-eye pathways and therefore may play a causal role.

### More Rapid Recovery with BV than with RO.

More than a decade ago, we showed that visual cortical responses to a contralateral eye deprived of vision for several days recover much more rapidly with BV than with RO in the mouse (37). This finding was initially surprising in light of the evidence from monkey studies, where BV was seen to be ineffective, and from the clinical experience with amblyopic patients, where “patch therapy” or some form of RO is the standard and generally effective therapy (38–40). However, a number of additional reports in various experimental animals confirmed that BV could be effective in restoring visual responses after MD and sometimes more effective than RO (41–48).

The reason for the difference between rodents on the one hand and human and non-human primates on the other need not be the underlying cellular neurobiology. Instead, the difference may be due to the dramatic imbalance in the mouse between the contralateral and ipsilateral visual pathways. In the mouse the contralateral-eye pathway provides 4 to 10 times as many inputs to even the most binocular portion of the visual cortex as does the ipsilateral-eye pathway, and the contralateral eye is much more effective than the ipsilateral eye in driving most cortical neurons. In the mouse, several days of contralateral-eye deprivation results in a visual cortex in which the two eyes are more or less equally effective. In humans and macaque monkeys, the strengths and numbers of inputs from the two eyes are similar to each other, and a similar period of MD makes the visual cortex nearly unresponsive to the deprived eye, leaving responses to be driven almost solely by the open eye. In the mouse, BV provides more or less twice the cortical activity as RO immediately upon eye opening. Because visual cortical responses to the deprived eye are almost absent in monkeys and humans, BV immediately upon eye opening stimulates about the same amount of cortical activity, and presumably BDNF production, as does RO.

While the present findings in mice on the superiority of BV over RO may have little direct relevance to the treatment of human amblyopia, the fact that BDNF production precedes and appears to be causal for the strengthening of a cortical pathway may indeed have great import for therapy. These findings raise the possibility that the application of an artificial activator of the TrkB receptor might prove a powerful adjunct to physical or cognitive therapy for brain injuries and disorders (reviewed in refs. (49–51).

## Methods

### Animals.

All procedures were approved by the Institutional Animal Care and Use Committee of University of California San Francisco. C57BL/6J wild-type mice were purchased from The Jackson Laboratories and bred in the UCSF animal care facility. TrkB^F616A^ mutant mice were as described (8, 9). Animals were kept in the standard housing condition with a 12-h light/dark cycle and a free access to food and water.

MD was performed by suturing the lid of the right eye (contralateral to the imaged hemisphere) at P25 as described (24). To examine recovery from MD, mice were imaged immediately before MD, after 5 d of MD, and at one of the time points (6, 12, 24, or 48 h) after restoring vision to the deprived right eye either by simply removing the suture (BV), or by reversing the lid suture (RO).

Continuous infusion of 1NMPP1 or vehicle solution was made into TrkB^F616A^ homozygous mice as described (9). Briefly, immediately after optical imaging of intrinsic signals on MD-5 d, an osmotic minipump (Alzet model 2001, Cupertino, CA) containing 1NMPP1 (0.25 nmol/ g body weight/ h) or vehicle solution was implanted subcutaneously. In addition, two intraperitoneal injections (16.6 ng/ g body weight) were made during the first 24 h of infusion to facilitate TrkB inactivation. Intracortical infusion of sTNFR or vehicle solution was performed using osmotic minipump (model 1002, Alzet) as described (24) during MD or BV. Briefly, a cannula was implanted into the medial edge of V1, immediately after intrinsic signal imaging of pre-MD baseline responses (for infusion during MD) or of post-MD responses (for infusion during BV). The cannula was connected to an osmotic minipump filled either with vehicle solution (PBS containing 0.1% BSA as a carrier) or 35 µg/mL of soluble TNF receptor-1 (sTNFR1, R&D Systems Inc.).

### Repeated Optical Imaging of Intrinsic Signals.

Repeated optical imaging of intrinsic signals and quantification of ocular dominance were performed as described (24). Briefly, during recording mice were anesthetized with 0.7% isoflurane in oxygen applied via a homemade nose mask, supplemented with a single intramuscular injection of 20 to 25 µg chlorprothixene. Images were recorded transcranially; the scalp was sutured closed at the end of each session and re-opened at the same location in subsequent sessions. Intrinsic signal images were obtained with a Dalsa 1M30 CCD camera (Dalsa, Waterloo, Canada) with a 135 × 50 mm tandem lens (Nikon Inc.) and red interference filter (610 ± 10 nm). Frames were acquired at a rate of 30 fps, temporally binned by 4 frames, and stored as 512 × 512 pixel images after binning the 1024 × 1024 camera pixels by 2 × 2 pixels spatially. The visual stimulus for recording the binocular zone, presented on a 40 × 30 cm monitor placed 25 cm in front of the mouse, consisted of 2°-wide bars, which were presented between −5° and 15° on the stimulus monitor (0° = center of the monitor aligned to center of the mouse) and moved continuously and periodically upward or downward at a speed of 10°/s. The phase and amplitude of cortical responses at the stimulus frequency were extracted by Fourier analysis as described (52). Response amplitude was an average of at least 4 measurements. Ocular dominance index (ODI) was computed as described (53). Briefly, the binocularly responsive region of interest (ROI) was chosen based on the ipsilateral eye response map after smoothing by low-pass filtering using a uniform kernel of 5 × 5 pixels and thresholding at 40% of peak response amplitude. We then computed OD score, (C−I)/(C+I), for each pixel in this ROI, where C and I represent the magnitude of response to contralateral and ipsilateral eye stimulation, followed by calculation of the ODI as the average of OD score for all responsive pixels.

The reliability of the quantitative use of intrinsic signal imaging for assessment of the strength of the responses to the two eyes has repeatedly been confirmed by showing that it provides a measure consistent with single unit recording in at least 6 papers that we have published since 2008 (24), and by 2-photon calcium imaging of the responses of single neurons (54).

### Western Blot Analysis.

The location of the binocular area in the primary visual cortex was identified based on the intrinsic signal map and the photograph of the surface blood vessels that were obtained before starting MD (Fig. 3*A*). The start time of recovery by BV or RO was adjusted so that all experimental groups were sampled at a similar time (early afternoon) to avoid possible circadian influences. The gray matter of binocular V1 contralateral to the initially-closed eye was dissected out under isoflurane anesthesia, frozen in liquid nitrogen, and stored at −80 °C. The samples from two animals of the same condition were pooled so that one lane in the blot contains two binocular V1. The tissues were homogenized with 5 volume of lysis buffer containing 50 mM Tris-HCl buffer (pH 7.5), 150 mM NaCl, 1 mM EDTA 1% Triton-X 100, 1% Na deoxycholate, 1% SDS, and protease inhibitor cocktail (Sigma, St. Louis), followed by centrifugation and collection of supernatants. Approximately 20 μg of total proteins in tissue lysates were separated on electrophoresis using 4 to 12% gradient SDS-polyacrylamide gels (BioRad) and transferred to the PVDF membrane (BioRad). After being blocked with 5% skim milk in 0.05% Tween-20/Tris-buffered saline (TBST), the membrane was incubated with polyclonal rabbit anti-BDNF (1:1,000 in TBST + 5% skim milk) at 4 °C for 2 h and then with horseradish peroxidase (HRP)-conjugated donkey anti-rabbit IgG (1:10,000 in TBST + 5% skim milk) at 4 °C for 1 h. The signals were detected using an enhanced chemoluminescence system (Amersham Biosciences) and visualized through X-ray film exposure (Kodak). The membranes were then stripped using Restore Western Blot Stripping Buffer (Pierce) for 4 to 6 h at room temperature and re-probed with anti-β actin antibody (1:2,000 in TBST + 5% skim milk) followed by HRP-conjugated donkey anti-goat IgG (1:15,000 in TBST + 5% skim milk). The exposed X-ray films were digitized and densitometric quantification was performed using ImageJ (National Institute of Health). The BDNF levels were normalized to corresponding β actin signals, and the deduced ratios were further normalized to that of the control ND mouse on the same blot to perform statistical analyses with ANOVA followed by Bonferroni’s post hoc test. Rabbit polyclonal anti-BDNF (N-20 sc-546) and goat polyclonal anti-β actin (C-11 sc-1615) antibodies were purchased from Santa Cruz Biotechnology. Secondary antibodies (donkey anti-rabbit IgG and donkey anti-goat IgG, HRP-conjugate) were from Jackson ImmunoResearch. All other chemicals were purchased from Sigma (St. Louis, MO) unless otherwise noted.

## Data Availability

All study data are included in the main text.
